# Prevalence of parasitic infections and associations with pregnancy complications and outcomes in northern Tanzania: a registry-based cross-sectional study

**DOI:** 10.1186/s12879-016-1413-6

**Published:** 2016-02-13

**Authors:** Aneth Mkunde Mahande, Michael Johnson Mahande

**Affiliations:** Department of Epidemiology & Biostatistics, Institute of Public Health, Kilimanjaro Christian Medical College, Moshi, Tanzania

**Keywords:** Prevalence, Parasitic infection, Pregnancy complication, Adverse pregnancy outcome

## Abstract

**Background:**

Parasitic infection(s) during pregnancy have been associated with increased risk of pregnancy complications and adverse outcomes in low resource settings. However, little is known about their influence on pregnancy outcomes. This study aimed to determine the prevalence of parasitic infections and their association with pregnancy complications and adverse outcomes.

**Methods:**

A retrospective cross-sectional study was conducted using maternally-linked data from Kilimanjaro Christian Medical Center (KCMC) medical birth registry. Birth records from all women who delivered singleton infants from 2000–2011 were utilized. We excluded multiple gestations and rural medical referral for various medical complications. A total of 30,797 births were evaluated. Data analysis was performed using SPSS version 18.0. Odds ratio (ORs) with 95 % confidence intervals (CIs) for adverse pregnancy outcomes and complications associated with parasitic infections were estimated using multiple logistic regression models. A *p*-value of less than 5 % was considered statistically significant.

**Results:**

The most prevalent parasitic infection recorded was malaria (17.0 %), while helminths and amebiasis were infrequently recorded (0.6 % vs. 0.7 %, respectively). Women who had malaria during pregnancy had 13 % increased odds of having a preterm delivery (OR = 1.13; 95 % CI: 1.01–1. 26) as compared to those who were not infected. They also had 33 % increased odds of getting maternal anemia (OR = 1.33; 95 % CI: 1.11–1.72). Likewise, pregnant women who were recorded with helminths infections had 29 % increased odds of having maternal anemia as compared to those who had no helminths infection (OR = 1.29; 95 % CI:0.48–3.53). Moreover, pregnant women who were recorded to have amebiasis had 79 % increased odds of having a preterm delivery as compared to those who had no ameba infection (OR = 1.79; 95 % CI: 1.12–2.91).

**Conclusions:**

Malaria was the prevalent parasitic infection in the studied population while helminth and ameba infections were infrequently reported. These parasitic infections were also associated with increased risk of anemia and delivery of a preterm infant. These were the only three infections/infestations which were evaluated. Our analysis revealed that malaria, helminth and ameba infections during pregnancy is dangerous and has life threatening consequences. This highlight the need to provide early diagnosis and treatment for infected women to prevent pregnancy complications and associated adverse pregnancy outcomes.

**Electronic supplementary material:**

The online version of this article (doi:10.1186/s12879-016-1413-6) contains supplementary material, which is available to authorized users.

## Background

Parasitic infections are common among pregnant women due to reduced body immunity and therefore can affect physiological systems of the body [1]. Malaria and ameba infections during pregnancy have been associated with increased risk of adverse pregnancy outcomes and complications [2, 3]. A recent study by Wekesa and colleagues in Kenya also found that intestinal helminth infestations during pregnancy was associated with greater risk of maternal complications and adverse perinatal outcomes such as anemia, low birth weight and perinatal mortality [4]. Previous investigators have demonstrated that, the effect of parasitic infections to a mother or an infant depends on the mother’s natural immunity, type of infecting parasite and parasitic load [5].

A multi-country study which was conducted among eight sub-Saharan African countries reported prevalence of malaria in pregnancy ranging from 10 to 65 % [6]. A systematic review which was conducted in sub-Saharan Africa estimated that 37.7 million women were infected with hook worm in 2005. Of these, 6.9 million were pregnant women. The report also showed that approximately 10 million women were infected with schistosomiasis [7]. In addition, amebiasis is the second leading cause of death among parasitic diseases after malaria [8]. It has been reported to infect about 10 % of the world’s population; and the prevalence may be as high as 50 % in tropical countries [9]. However, estimation of amebiasis during pregnancy remains a challenge due to problems associated with proper diagnostic distinction between the pathogenic form *(Entamoeba histolytica)* and the non-pathogenic *(Entamoeba Dispar)* [10].

A study by Kalilani and colleagues reported on the association between parasitic infections during pregnancy and increased risk of multiple complications such as maternal anemia, miscarriage, intrauterine growth retardation and maternal death [3]. In addition, parasitic infections may increase the risks of low birth weight, preterm delivery, stillbirth, fetal anemia and fetal mortality [11]. Previous studies conducted in sub-Saharan Africa including Tanzania on this topic used cross-sectional design with small sample size and focused on malaria, helminths or schistosomiasis among non-pregnant women or school children. A previous study on parasitic infection among pregnant women was based on fairly small sample size (<500 subjects) [12], which mainly studied on the relationship between parasitic infections and maternal anemia [13].

This study aimed to determine the prevalence of parasitic infections and its association with pregnancy complications and adverse outcomes. Better understanding of this relationship is crucial in the development of effective strategies for reducing adverse maternal and fetal outcomes and complications related to parasitic infections.

## Methods

### Study design

A retrospective cross-sectional study was conducted using maternally-linked data from KCMC medical birth-registry. Birth records from all women who delivered singletons for the period from 2000–2011 were utilized.

### Study setting

This study was conducted at KCMC which is one of four general referral hospitals in Tanzania, situated in Moshi urban district (municipal) of Kilimanjaro region in Northern Tanzania. The hospital receives deliveries from nearby communities and referral cases from other health care facilities inside the region and the nearby regions. The hospital has an average annual delivery rate of 4000 births.

### Study participants & sample size

**Fig. 1:**
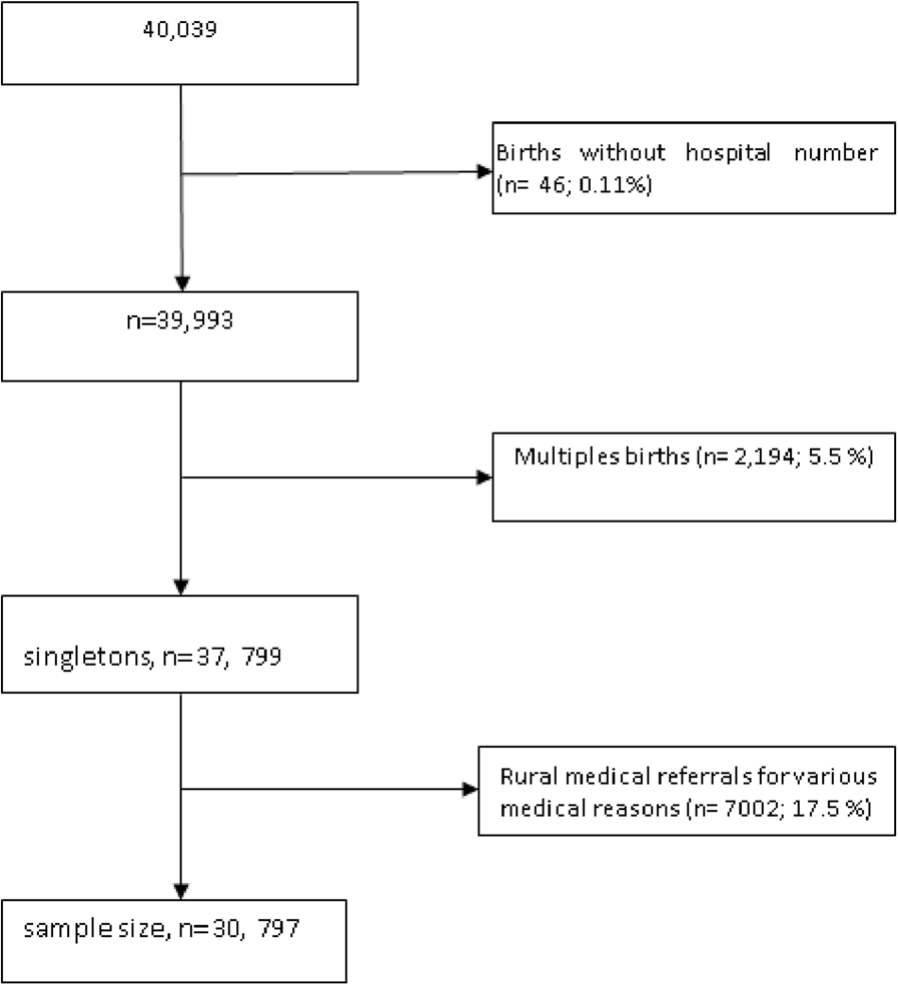
Flow chart showing the numbers of participants

From 2000 to the end of year 2011, a total of 40,039 deliveries were recorded at the medical birth registry. We excluded multiple gestations and rural medical referral for various medical complications which were not related to parasitic infections to avoid overrepresentation of studying women with high risk pregnancies. The remaining 30,797 births which constituted our sample size were evaluated (Fig. 1).

### Study variables

In this study, the pregnancy complication such as maternal anemia and pregnancy outcomes (i.e. preterm delivery, low birth weight, perinatal death and birth defects) were the main outcomes.

Gestational age at birth was calculated from the first day of the woman’s last menstrual period and counted in completed weeks. Low birth weight was defined as infant born with a weight of less than 2500 grams while Preterm birth was defined as birth occurred at less than 37 weeks gestation. Perinatal mortality was defined as the number of stillbirths and deaths in the first week of life (early neonatal deaths) per 1000 live births.

Birth defects which were recorded in the birth registry included; hydrocephalus, spinal bifida, anencephaly (neural tube defect), tallipes, potters syndrome, malformation of the legs, neck malformation, congenital heart disease, congenital keloids and conjoined twins.

The exposure/predictor variables in this study include major parasitic infections as recorded in the medical birth registry i.e. malaria, helminthes (intestinal worm & schistosomiasis) and amebiasis infection. The socio demographic variables studied includes maternal age, maternal occupation, marital status, antenatal care visit (ANC) and area of residence. Mother occupation variable was categorized in two groups; employed (professional and service) and unemployed (housewife, farmer, business, student). Education variable was categorized into “lower education” (<12 years) is up to 11 years of schooling and “upper education” ( ≥12) from 12 years of schooling to higher education.

We also analyzed hygiene and sanitation variables including mothers who were drinking boiled water, water source (tap water, well, river, spring and others) and type of toilet (toilets with flush, pit latrines).

Some maternal sociodemographic factors such as maternal age, parity, education, occupation, marital status and antenatal care visits were considered as potential confounders. While maternal HIV status was considered as an effect modifier for the association between malaria and anaemia.

### Data collection (Data source)

We used secondary medical birth registry data which was prospectively collected through interview using a standardized questionnaire to all women who delivered at KCMC for the period of 2000 to 2011. Each woman was individually consented to participate prior the interview. According to medical birth registry regulations, each woman who gives birth at KCMC is assigned with a unique identification number (ID) which is constant for all births that occur at KCMC. The mother ID is also assigned to her respective siblings; this makes it possible to create a reproductive history for each woman for subsequent births that occurred at KCMC. During the interview, each woman was asked if she had any parasitic infection during pregnancy (self-reported by the woman). Mothers were also asked to bring their antenatal care visit card to validate some information with regard to pregnancy status including infections. All the information collected was recorded and entered in the computerized database at the medical birth registry. In summary, the information collected from women includes maternal and paternal sociodemographic characteristics, the health status of the mother before pregnancy, during pregnancy (including a parasitic infection which was recorded as infections during pregnancy) and delivery and infant status after birth (see Additional file 1).

### Bias

Selection bias is a common problem in hospital based studies. For example, women with pregnancy complications during prenatal care are more likely be advised by their clinician or care providers to have delivery at tertiary hospitals for delivery. This may lead to overrepresentation of high risk women who deliver at tertiary hospital which may result to overestimation of the studied adverse perinatal outcomes. On the other hand, the effect of parasitic infections on pregnancy outcomes depends on gestational age when the infections occur, Due to lack of routine screening for parasitic infection among pregnant women in the study setting, majority of women had diagnosis for parasitic infections during their second or third trimesters. This may lead to underestimation of the reported prevalence of parasitic infections.

### Ethical consideration

The ethical approval was obtained from Kilimanjaro Christian Medical University College Research and Ethics Committee (KCMUCo) for the registry project. Both written and verbal consents were sought from individual mothers prior to the interview. Since there was no participant aged less than 16 years, each participant was able to provide an informed consent. Participants were also informed about the importance of the medical birth registry project, and that participation to the study was on voluntary basis. Both confidentiality and privacy were adhered to, where use of unique mother identification number was used instead of names and interviews were conducted in a privacy room.

### Statistical analyses

Data analysis was performed using SPSS Software (SPSS Inc., Chicago, IL) version 18.0 for analysis. Student t-test was used to compare the difference in means for continuous variables The Chi-squared test (χ^2^) was used to compare proportions of categorical variables. The odds Ratios (ORs) with 95 % confidence intervals for pregnancy complications and adverse pregnancy outcomes were estimated using multivariable logistic regression model while controlling for potential confounders. Five models were run where each adverse pregnancy outcome (i.e. preterm birth, low birth weight, perinatal death and birth defect) and complication (i.e. maternal anaemia) were treated as an outcome and assessed independently in a multivariable logistic regression model against parasitic infections (malaria, helminths and amoeba) as an independent variables (Table 3).

A variable was considered to be a confounder if its inclusion in the model changed the crude odds ratio by 10 % or more. We also tested an interaction between malaria infection and HIV on anaemia, but we found that there was no statistical intercation.

## Results

### Prevalence of parasitic infections

A total of 30,797 births were evaluated. There were only three infections/infestations which were evaluated including malaria, helminthiasis and amebiasis. The prevalence for malaria, amebiasis and helminthiasis was 17 %, 0.7 % , and 0.6 % respectively.

### Association between sociodemographic characteristics and parasitic infections

The association between sociodemographic characteristics and prevalence of parasitic infections are shown in Table 1. Malaria, helminthiasis and amebiasis were significantly associated with most of the demographic variables studied (Table 1), except for amebiasis which not dependent on maternal education level, type of toilet and area of residence. We found that number of antenatal care visits (>4 visits), being unemployed, low maternal education attainment (<12 years), residing in rural area were associated with an increased risk of helminthesis and amebiasis.

**Table 1:** Association between sociodemographic characteristics and parasitic infections

		Parasitic infections		
	Total (*n* = 30,797)	Malaria (*n* = 30,411)	Helminths (*n* = 181)	Amebiasis (*n* = 205)
Characteristics	*N*	%	%	%
Age (years)				
< 20	2182	13.1	1.0	0.3
20-34	24072	17.7	0.5	0.7
≥ 35	4306	16.5	0.6	0.9
χ^2^ *p* value		<0.001	0.03	0.024
ANC visits				
< 4 times	9128	14.5	0.4	0.5
≥ 4 times	20379	18.0	0.6	0.7
Missing	1290			
χ^2^ *p* value		<0.001	0.03	0.03
Occupation				
Unemployed	22751	16.2	0.6	0.6
Employed	7865	19.9	0.4	0.8
Missing	181			
χ^2^ *p* value		<0.001	0.03	0.21
Education				
< 12	19588	0.16	0.7	0.7
≥ 12	11192	0.19	0.4	0.6
Missing	17			
χ^2^ *p* value		<0.001	<0.01	0.51
Area of residence				
Rural	11367	17.4	0.9	0.7
Urban	19344	17.0	0.4	0.6
Missing	86			
χ^2^ *p* value		0.39	<0.001	0.42
Boiling water				
No	6849	23.1	1.0	1.0
Yes	23835	15.5	0.6	0.5
Missing	113			
χ^2^ *p* value		<0.001	<0.001	<0.001
Water source				
Others	1853	19.6	1.2	1.3
tap water	28797	17.0	0.5	0.6
Missing	148			
χ^2^ *p* value		0.005	<0.001	<0.001
Type of toilet				
Pit-latrine	16565	16.4	1.2	0.7
Flush	14163	18.1	0.5	0.6
Missing	69			
χ^2^ *p* value		<0.001	<0.001	0.39

*P*value calculated from chi square test

### Sociodemographic characteristics in relation to maternal anemia and adverse pregnancy outcomes

The sociodemographic characteristic associated with adverse pregnancy outcome and maternal anemia are presented in Table 2. Poor attendance to antenatal care (<4 visits) was significantly associated with preterm delivery (*P* < 0.001). Furthermore, extreme maternal age (<20 and ≥35 years), primiparity (i.e. para 1), being unmarried, being unemployed, having low education, positive HIV status and living in rural area were also significantly associated with delivery of low birth weight infant (*P* < 0.05). We also found a significant increase in perinatal mortality in relation to high maternal age (≥35 years), poor attendance to ANC, unemployed, low education and living in rural area. On the other hand, low maternal age (<20 years) and primiparity were significantly associated with maternal anaemia *P* < 0.05).

**Table 2:** Sociodemographic associated with adverse pregnancy outcome and maternal anemia

		Adverse pregnancy outcomes and maternal anemia				
Characteristics	Total	Preterm birth	LBW	Perinatal death	Birth defect	Anemia
	*N* = 30,797	(*n* = 3,345)	(*n* = 2,856)	(*n* = 1,038)	(*n* = 48)	(*n* = 518)
Age group	N	%	%	%	%	%
< 20	2210	11.0	10.6	2.9	0.1	2.4
20–34	24237	10.9	9.0	3.2	0.1	1.7
≥ 35	4350	11.0	10.1	4.6	0.3	1.5
χ^2^ *p* value		0.97	0.006	<0.001	0.2	0.03
Parity						
1	9508	13.0	10.1	2.6	0.1	2.2
2	7392	13.0	8.3	3.1	0.2	2.0
≥ 3	8528	13.3	9.6	4.6	0.2	1.6
Missing	5369					
χ^2^ *p* value		0.87	<0.001	<0.001	0.4	0.01
ANC visits						
< 4	9128	6.6	15.2	4.5	0.2	1.6
≥ 4	20379	12.5	6.5	2.7	0.1	1.7
Missing	1290					
χ^2^ *p* value		<0.001	<0.001	<0.001	0.7	0.8
Marital						
Single	3331	11.0	12.2	3.2	0.1	1.6
Married	27343	11.0	8.9	3.3	0.2	1.7
Missing	123					
χ^2^ *p* value		0.9	<0.001	0.62	0.6	0.8
Occupation						
Unemployed	22751	10.8	9.7	3.5	0.2	1.7
Employed	7865	11.3	8.2	2.9	0.1	1.7
Missing	181					
χ^2^ *p* value		0.19	<0.001	0.01	0.7	0.80
Education						
< 12	19588	11.1	10.1	3.7	0.2	1.7
≥ 12	11192	10.7	8.0	2.8	0.1	1.6
Missing	17					
χ^2^ *p* value		0.32	<0.001	<0.001	0.10	0.7
HIV status						
No	20248	5.7	5.5	5.7	5.8	5.8
Yes	1244	6.4	8.3	7.4	12.5	6.9
Missing	9305					
χ^2^ *p* value	0.2	0.2	0.01	0.08	0.3	0.4
Area of residence						
Rural	11367	12.1	10.4	4.2	0.2	1.9
Urban	19344	10.3	8.6	2.9	0.1	1.6
Missing	86					
χ^2^ *p* value		0.14	0.001	<0.001	0.002	0.08
Overall prevalence		10.9	9.3	3.4	0.2	1.7

### Association between parasitic infections with adverse pregnancy outcomes and maternal anemia

The results from multivariable logistic regression models for association between maternal anemia, pregnancy outcomes and parasitic infections are shown in Table 3. Since the binary logistic regression model is suitable for binary outcome and we have five outcomes; we performed five models where each adverse pregnancy outcome and complication was treated as an outcome while parasitic infections (i.e. malaria, schistosomiasis and helminths) were treated as an exposure variables for each model. Since some demographic characteristics were independently associated with outcome of interest, we adjusted for maternal age, parity, antenatal care visits, maternal education, maternal occupation, area of residence and marital status. Women recorded with malaria infection during pregnancy had 33 % (OR 1.33, 95 % CI: 1.07–1.66) increased odds of getting anemia as compared to those who were not infected. These women were also 1.13 times (95 % CI: 1.01–1.26) more likely to have preterm delivery. Malaria infection during pregnancy was also associated with increased odds of birth defect (OR 1.49, 95 % CI: 0.73–3.02), but this association was not statistically significant.

**Table 3:** Association between parasitic infections with pregnancy outcomes and complications

	Adverse pregnancy outcomes and maternal anemia				
	OR (95 % CI)				
Parasitic infections	Model 1	Model 2	Model 3	Model 4	Model 5
	Preterm birth	Low birth weight	Perinatal death	Birth defect	Anemia
Malaria	2.51 (2.43–2.72)	1.10 (0.94–1.13)	1.03 (0.91–1.21)	1.42 (0.71–2.83)	1.51 (1.21–1.83)
Malaria^a^	1.12 (1.01–1.26)	1.05 (0.95–1.17)	1.04 (0.86–1.24)	1.49 (0.73–3.02)	1.33 (1.07–1.66)
Helminths	0.95 (0.59–1.54)	1.15 (0.72–1.86)	1.33 (0.65–2.71)	0.94 (0.11–7.88)	1.67 (0.68–4.07)
Helminths^a^	0.71 (0.42–1.19)	1.07 (0.62–1.84)	0.70 (0.26–1.91)	(-)	1.29 (0.48–3.53)
Amebiasis	1.54 (1.05–2.25)	1.21 (0.72–2.01)	0.72 (0.31–1.74)	3.21 (0.42–23.13)	0.62 (0.12–2.34)
Amebiasis^a^	1.79 (1.12–2.86)	0.26 (0.15–0.47)	0.25 (0.12–0.64)	1.37 (0.13–14.13	0.41 (0.09–1.77)

^a^Adjusted for maternal age, parity, antenatal care visits maternal education, maternal occupation, area of residence, marital status. (-): The adjusted odds ratio for the association between helminths infection and birth defect was not computed because of fewer cases of birth defect among women who had helminths infection

We also found that women who were recorded to have helminth infestations during pregnancy had 1.3-fold (95 % CI: 0.48–3.53) increased odds of maternal anemia as compared to their counterparts who were not infected, but this association did not reach statistical significance. Women recorded with amebiasis were more likely to deliver preterm infants as compared to women who were not infected (OR 1.79; 95 % CI: 1.12–2.9).

Since malaria and HIV both have an influence on maternal anaemia, we performed a sub-analysis to test for interaction between malaria and HIV co-infection on maternal anemia, but there was no statistically significant interaction (data not shown).

## Discussion

In this study, malaria was the commonest parasitic infection among pregnant women in the study population while amebiasis and helminthiasis were infrequently reported. Malaria was also associated with increased odds of maternal anaemia and preterm birth while amebiasis infections in pregnancy increased the odds of preterm birth.

The prevalence of malaria in our study was consistent with 16.4 % that was reported in the northwest of Tanzania [11] and 18 % in Kenya [14]. But it was lower than 37 % and 58 % that was reported in Congo and Ghana [1, 15]. It was however higher than 9.5 % that was previously reported in Tanzania [16]. The difference in prevalence may be explained by the differences in endemicity of parasitaemia which might be attributed to behavioural and environmental exposure to malaria.

The prevalence of helminth infestation observed in our study was lower as compared to 17.6 % and 25.7 % that was reported in Ghana [17, 18]. and 17.4 % in Guatemala [19]. On the other hand, the prevalence of amebiasis in our study was also lower compared to previous studies [20–23]. The difference in prevalence of these infections could be explained by the differences in the studied population and nature of study design. The previous studies used prospective cohort design where it was possible to capture this infection across the gestational age period unlike for the present study. Another possible explanation for the lower prevalence of these infections may be due poor recall of the infection as women were asked retrospectively, which may lead to underestimation of the reported prevalence of infections.

Furthermore, women with high number of antenatal care visits were more likely to have parasitic infections as compared to those who had less than four visits. This could be a reflection of being over diagnosed during the ANC visits as women who attend the recommended number of ANC visits are more likely to be diagnosed for various infections/infestations across the gestational period than those with less ANC visits, which is also similar to previous investigators [21].

A previous study in Tanzania found that malaria infection during pregnancy contributed 15 % of maternal anemia [24]. In line to this study, pregnant women who were infected with malaria had 33 % increased odds of developing anemia as compared to those who were not infected. Our result also corresponds with previous studies conducted in Sub-Saharan Africa [6, 15, 25, 26]. The reason for difference in prevalence between our study and the previous study in Tanzania could be explained by the differences in the population studied. The former used population based data which may comprise low risk population.

The pathogenesis of anemia by malaria parasites (*P.falciparum*) includes the hemolysis of the infected red blood cells, reduction in production of red blood cells and other possible causes for instance removal of uninfected red blood cells due to antibody sensitization, and rupture of infected red blood cells with pathological effects of accompanying actions. Marrow hypoplasia occurs in acute infections of malaria which may reduce the production of red blood cells [27]. The mentioned processes may also explain the observed high odds of anemia in the present study.

Our results showed that women infected with helminths had higher odds of having maternal anemia, although this association did not reach statistical significance probably due to small number of cases with helminth infestation. This finding is in line with previous reports [13, 28, 29].

The prevalence of preterm birth in our study is consistent with the National preterm rate of 12 % [24]. We found women who had malaria infection during pregnancy were more likely to have preterm delivery. This finding is in agreement with previous studies in Malawi [30] and Tanzania [16].

The mechanism by which malaria can increase the risk of preterm birth is well known. Evidence suggests that *P.falciparum* infection in a pregnant woman is characterized by sequestration of the infected erythrocytes in the maternal placental vascular area [27]. This may cause generation of an immune response which is characterized by monocytic infiltrates in the intervillous space of placenta and change in cytokine balance which may cause preterm delivery.

In the present study we found that women who were infected with malaria during pregnancy were less likely to deliver an infant with low birth weight. Similar observation was reported elsewhere [31]. Lack of association between malaria infection and low birth weight in our study may be explained by the timing and frequency of *P.falciparum* infection during pregnancy. Therefore, it may be possible that the majority of women in the present study may had malaria infection during their third or first trimester which have been reported to have little effect on fetus birth weight than the second trimester which is the period of maximum growth of the fetus [3].

We found no association between malaria infection and perinatal mortality. A similar finding was reported previously in Tanzania by Hinderaker et al. [32]. Lack of association between malaria and perinatal death in our study and that of others could be explained by improved quality of health care service in the studied areas.

In this study we found no significant association between helminths infections and risk of giving birth to low birth weight infant. Similarly finding was reported in Kenya by Fairley and his colleagues [33]. But in contrast with a study in Asia which reported high odds of low birth weight among infants born to women who had helminth infestation during pregnancy [31]. The difference in findings between our study and that of others could be attributed to differences in the studied populations. We did not find any significant association between helminths infection during pregnancy and preterm birth, perinatal death or birth defects. Our results are in line with previous study which was conducted in South East Asia [31].

Amebiasis during pregnancy is thought to be more severe than in non-pregnant [34]. In the present study women with amebiasis infection had 2-fold increased odds of preterm delivery compared with the reference group. Pregnant women are susceptible to penetration of intestinal mucosa and placental barrier especially by *Entamoeba histolytica*. The immunocompromization during pregnancy can lead to reduced production of IgA which may lead to sub-chronic inflammation and placenta dysfunction which has been associated with preeclampsia, indicated preterm delivery and fetal growth restriction [35]. This could explain the observed finding in our study. However, the etiological mechanism for amebiasis to cause chronic inflammation needs further investigation.

### Strengths and limitations of the study

Our study used a large sample size which makes it possible to make an inference with high precision. Information was collected by well-trained nurse’s midwives through interviewing the women using a standardized questionnaire and supplemented with information obtained from medical records and antenatal care clinic cards to confirm the information from the questionnaire to ensure data completeness and accuracy for most important study variables.

Our study also had some limitations which are important to be taken into account while interpreting this result. First, this study used hospital-based data which have already been recorded for performing different research projects and not specifically meant for this study. Secondly, the assessment of parasitic infection was based on self-reported information which may lead to under or overestimation of the reported prevalence of infection. Third, this was a hospital-based study which may suffer from referral bias. However, this may have huge consequences in generalization of our findings if women who were studied had different risk characteristics compared to other women in the general population. We excluded women who were referred from rural facilities for various medical complications and those with multiple gestations to avoid overrepresentation of high risk women in our study. The effect of this exclusion on results is not known. But we believe that the exclusion of these women was not related to severe parasitic infections. Fourth, we did not assess the effect of other maternal variables which have been associated with adverse perinatal outcomes such as interpregnancy interval, preeclampsia, delivery complications as well as missing data. The effect of these factors on the studied outcomes remains unclear. Fifth, we used data that were collected for many years, therefore some diagnosis criteria for studied parasitic infections may have been changed over time; this may affect the reliability of the reported findings. Finally, Data on parasitic infections were based on self-reported information by participants; this may lead to self desirability bias and compromise validity of our findings.

## Conclusion

Our finding adds to the evidence of adverse health effects of parasitic infections on pregnancy complications and outcomes studied. Malaria in pregnancy was found to be an important risk factor for anemia as well as contributing to increased risk of preterm birth while amebiasis infections in pregnancy increased the risk of preterm birth. In addition, some factors related to water quality and sanitation were found to be associated with helminthiasis and amebiasis particularly among the rural women. The prevalence of amebiasis and helminthiasis was very low and probably does not reflect the reality due to underreporting of infection/infestation. Our findings suggest that it is very important that pregnant women with malaria and amebiasis to be offered with early diagnosis and adequate treatment during pregnancy to prevent adverse pregnancy outcomes and complications. Further community based research using proper diagnostic procedures are needed to confirm our findings.

## Additional files

Additional file 1:
**KCMC Medical Birth Registry.** (DOCX 404 kb)
