# Supplementary material for: Prevalence of parasitic infections and associations with pregnancy complications and outcomes in northern Tanzania: a registry-based cross-sectional study
Source: BMC Infect Dis. 2016 Feb 13;16:78. doi: 10.1186/s12879-016-1413-6 (PMC4753041; doi:10.1186/s12879-016-1413-6)
Supplement: Additional file 1: — KCMC Medical Birth Registry. (DOCX 404 kb) [file 12879_2016_1413_MOESM1_ESM.docx]

KCMC Medical Birth Registry

**1 Basic information concerning mother**

**1.1 Mothers date of birth: Age: 1.2 Mothers name:**

**1.4 Hospital number: 1.3 Address:**

**1.6 Birth number: 1.5 Date of interview:**

**1.8 Date of admission: 1.7 Interview by:**

**Referred for delivery:** 1 Yes If yes: ⇒**Referred from: Referred during labour:**

2 No (self referral) 1 Home 1 Admitted in labour

Reason for referral: 2 Regional hospital 2 Admitted before labour

3 District hospital

4 Other, specify:

**1.9 1.10 Date leaving hospital:**

**Official date of discharge:**

**1.11 Current residence:** 1 Rural **1.12 Mothers childhood** 1 Rural

2 Urban **residence:** 2 Urban

3 Semi urban 3 Semi urban

Area of mother's Area of mother's childhood

residence: residence:

**1.13 Highest educational level:** 1 None **1.14 Current occupation:** 1 Housewife

2 Primary (1-7) 2 Farmer

3 Secondary (8-11) 3 Service

4 Higher (12+) 4 Business

5 Professional

**1.15 Current marital status:** 1 Married Age at first marriage: 6 Student

2 Single 7 Others ⇒

3 Widowed

4 Remarried **No of previous**

5 Divorced **pregnancies**

6 Polygamous family Add wife number:

**If yes, at age: If yes, type:**

**1.16 Regular menstrual** 1 Yes Age at **1.17 Genital** 1 Yes 1 Type one

**periods:** 2 No menarche: **mutilation** 2 No 2 Type two

***(Circumcision)***

3 Type three

**1.18 Mother’s tribe:** 01 Chagga **1.19 Religion:** 1 Catholic 4 Other types

02 Pare 2 Protestant

03 Masai 3 Muslim

Other ⇒ 4 Others ⇒

**2 Questions concerning the father of the child:**

**2.1 2.2**

**Father’s name**: **Father’s age:**

**2.3 Current occupation of father: 2.4 Father’s** 1 None

01Farmer 06 Official **educational level:** 2 Primary (1-7)

02Business 07 Professional 3 Secondary (8-11)

03Skilled worker 08 Student 4 Higher (12+)

04Unskilled worker 09 Unemployed **2.5 Father’s tribe:** 1 Chagga

05Service 10 Other ⇓ 2 Pare

3 Masai

4 Others ⇒

**3 Questions concerning home conditions:**

**3.1 Source of drinking water:** 1 Tap water **3.3 Distance to water, if not** 1 Less than 1 km (less ½ hour walk)

2 Well **tap:** 2 More than 1 km,

3 River specify in km:

4 Spring **3.4 Home toilet:** 1 Pit latrine

5 Other, specify 2 Flush

**3.2 Boiling of drinking water**: 1 Yes 3 Others ⇒

2 No

4 **Mothers health before and during present pregnancy**

**4.1 Body weight (kg): 4.2 Body height (cm): 4.3 Blood** 1 Yes

(before pregnancy) **transfusions** 2 No

**4.4 Serious** 01 Diabetes 06 Anaemia 11 Tuberculosis

**diseases** 02 Hypertension 07 Gynaecological disease 12 Sickle cell

03 Heart diseases 08 Liver disease (jaundice) 13 Other, specify ⇓

04 Epilepsy 09 Kidney disease

05 Malaria 10 Lung disease

**4.5 Have you ever practised** 1 Yes Months trying to get pregnant:

**family planning:** 2 No

If yes, what kind of 01 Pills 05 Implant 09 Abstinence

prevention 02 Injections 06 Lactation 10 Traditional

03 IUD 07 Withdrawal 11 Other specify ⇓

04 Condoms 08 Natural

**4.6 Antenatal care in** 1 Yes If yes: First medical appointment date:

**this pregnancy:** 2 No

If date unknown, estimate 1 0-12. week of gestation

Number of visits: first appointment : 2 13-20. week

3 21-30. week

4 After 31. week

**4.7 L.M.P: 4.8 Ultrasound** 1 Yes **4.9 E.D.D. based on**

2 No **clinical estimate:**

**4.10 Do you smoke?** 1 Yes If yes: how many Smoking during this pregnancy: 1 Yes

2 No cigarettes per day: 2 No

1 1

Chewing tobacco Yes Chewing tobacco during this Yes

2 No pregnancy: 2 No

**4.11** Do you drink alcoholic 1 Yes Did you also drink alcoholic 1 Yes

beverages? 2 No beverages during this pregnancy? 2 No

If yes: 1 Every day If yes: 1 Every day

2 More than once a week 2 More than once a week

3 Once a week 3 Once a week

4 Occasionally 4 Occasionally

**4.12**

**Drugs on regular** 1 Yes ⇒ Did you take any drugs during 1 Yes ⇒

**basis?** 2 No this pregnancy: 2 No

If yes: 1 Modern If yes, specify: 1 Modern

2 Traditional 2 Traditional

**Did you take any drugs** 1 Yes ⇒ Drugs for infertility: 1 Yes

**at time of conception or** 2 No 2 No

**during first trimester:**

**4.15 Blood Rh:** Anti-D in previous 1 Yes **VDRL status** 1 Positive

**group (AB0)** pregnancies: 2 No 2 Negative

Hb measurement done: 3 Unknown 3 Unknown

**Hb** 1 On Admission

2 Last visit to ANC

Specify

**HIV test recorded** 1 Yes ⇒ If yes, result:. 1 Negative Treatment during this 1 Yes

2 No 2 Positive pregnancy: 2 No

**4.16 Diseases and** 1 Yes (specify below) 06 Epilepsy 13 Gynaecological disease

**complications** 2 No 07 Bleeding 14 Tromboembolic disease

**during present**

08 Anaemia 15 Heart disease

**pregnancy,**

01 Gestational diabetes 09 Hyperemesis 16 Tuberculosis

**including accidents:**

02 Diabetes 10 Malaria 17 Lung disease

03 Hypertension 11 Jaundice 18 Infections, specify

20 Preeclampsia, mild 12 Schistosomiasis 19 Others, specify 🙖

21 Preeclampsia, severe

05 Eclampsia

**5 Questions concerning the delivery**

**5.1 At birth** 1 Single birth If multiple, add **Weight on 5.2 Complications** 1 PROM

2 no. of children: **admission: during delivery** 2 Bleeding > 500 ml

Multiple birth⇒

3 3-4. degree tear

**5.3 Induction of** 1 Yes If yes: 1 Amniotomy **5.4 Others** 1 Episotomy 4 Abruption of placenta

**labour** 2 No 2 Oxytocin 2 Symphysiotomy 5 Placenta previa

3 Prostaglandin 6 Other complications

**5.5 Analgesia:** 1 Yes Specify type other type of complication

2 No

**5.8 Blood Loss** (ml)

**5.6 Anaesthesia:** 1 General

2 Spinal/Epidural **5.9 Mother’s health** 1 Good Cause of death:

**after delivery** 2 Fair **Post mortem:**

**5.7 Gestational age at birth** 3 Bad 1 Yes

**clinical estimate** 4 **Maternal death** 2 No

**6 Status of 1. child (Always fill inn)**

**6.1 Date of delivery 6.3 Sex** 1 Male **6.4 Birth weight**

2 Female (gram)

**6.2 Time of delivery** 3 Unknown, unspec. **6.5 Length 6.6 Head**

(cm) **circum**

**6.7 Presentation:** 1 Cephalic **6.8 Status** 1 Live born

2 Breech 2 Live born transferred to paediatrics dept

3 Transverse 3 Stillborn Cause of death

4 Other 4 Neonatal death

**6.9 If stillborn:** 1 Dead before labour **If stillborn, also specify: And: Post mortem:**

2 Dead during labour 1 Dead before admission 1 Fresh 1 Yes

3 Unknown, unspec. 2 Dead after admission 2 Macerated 2 No

**If neonatal** 1 Died within first 24 hours Date of death:

**6.10 Apgar 1min 5 min 10 min**

**death:** 2 Died within first week

**score:**

**6.11 Mode of** 1 Spontaneous 4 CS elective Indication when Primary

**delivery:** 2 Vacuum, vaginal 5 CS others caesarean section: Secondary

3 Forceps, vaginal 6 Assisted breech **6.12 Failed intervention** 1 Vacuum

7 Destructive operative 2 Forceps

**6.13 Does the child have** 1 Birth defects

**any of these** 2 Injuries

**conditions?**

3 Diseases

4 HIV Positive

**Status on 2. child (For multiple births – not for singletons, if more than twins add extra copy of this page)**

**6.1 Date of delivery 6.3 Sex** 1 Male **6.4 Birth weight**

2 Female (gram)

**6.2 Time of delivery** 3 Unknown, unspec. **6.5 Length 6.6 Head**

(cm) **circum**

**6.7 Presentation:** 1 Cephalic **6.8 Status** 1 Live born

2 Breech 2 Live born transferred to paediatrics dept

3 Transverse 3 Stillborn Cause of death

4 Other 4 Neonatal death

**6.9 If stillborn:** 1 Dead before labour **If stillborn, also specify: And: Post mortem:**

2 Dead during labour 1 Dead before admission 1 Fresh 1 Yes

3 Unknown, unspec. 2 Dead after admission 2 Macerated 2 No

**If neonatal** 1 Died within first 24 hours Date of death:

**6.10 Apgar 1min 5 min 10 min**

**death:** 2 Died within first week

**score:**

**6.11 Mode of** 1 Spontaneous 4 CS elective Indication when Primary

**delivery:** 2 Vacuum, vaginal 5 CS others caesarean section: Secondary

3 Forceps, vaginal 6 Assisted breech **6.12 Failed intervention** 1 Vacuum

7 Destructive operative 2 Forceps

**6.13 Does the child have** 1 Birth defects

**any of these** 2 Injuries

**conditions?**

3 Diseases

4 HIV Positive
